# Diagnostic, clustering, and immune cell infiltration analysis of m6A regulators in patients with sepsis

**DOI:** 10.1038/s41598-022-27039-4

**Published:** 2023-02-13

**Authors:** Fenghui Li, Yuan Zhang, Zhiyun Peng, Yingjing Wang, Zhaoshang Zeng, Zhongxiang Tang

**Affiliations:** Intensive Care Unit, Affiliated Guangdong Hospital of Integrated Traditional Chinese and Western Medicine of Guangzhou University of Chinese Medicine, Foshan, 528000 Guangdong Province China

**Keywords:** Computational biology and bioinformatics, Biomarkers, Medical research, Risk factors

## Abstract

RNA N6-methladenosine (m6A) regulators are required for a variety of biological processes, including immune responses, and increasing evidence indicates that their dysregulation is closely associated with many diseases. However, the potential roles of m6A regulators in sepsis remain unknown. We comprehensively analyzed the transcriptional variations in and interactions of 26 m6A regulators in sepsis based on the Gene Expression Omnibus (GEO) database. A random forest (RF) model and nomogram were established to predict the occurrence and risk of sepsis in patients. Then, two different m6A subtypes were defined by consensus clustering analysis, and we explored the correlation between the subtypes and immune cells. We found that 17 of the 26 m6A regulators were significantly differentially expressed between patients with and without sepsis, and strong correlations among these 17 m6A regulators were revealed. Compared with the support vector machine (SVM) model, the RF model had better predictive ability, and therefore was used to construct a reliable nomogram containing 10 candidate m6A regulators to predict the risk of sepsis in patients. In addition, a consensus clustering algorithm was used to identify two different subtypes of m6A, which helped us distinguish different levels of immune cell infiltration and inflammation in patients with sepsis. Comprehensive analysis of m6A regulators in sepsis revealed their potential roles in sepsis occurrence, immune cell infiltration and inflammation in patients with sepsis. This study may contribute to the development of follow-up treatment strategies for sepsis.

## Introduction

Sepsis is a life-threatening organ dysfunction resulting from a dysregulated host response to infection^1,2^. It is estimated that 90% of global deaths from infection occur in Asia and Africa, and approximately 70% of the 9 million newborn and infant deaths from infection have been linked to sepsis^3^. Timely diagnosis and risk estimation are the key to reducing sepsis complications and mortality^4^. With the emergence of precision medicine research, genomics and other omics technologies have made great progress at the molecular level, an advance that also provides the possibility of precise treatment strategies for sepsis.

Previous studies have attempted to identify different subtypes of sepsis by clinical features or biomarkers. Molecular diagnostic and risk stratification were performed based on genome-wide blood gene expression profiles, and four molecular subtypes of sepsis were identified^5^. Davenport et al. used unsupervised clustering to analyze the overall gene expression of peripheral leukocytes in community-acquired pneumonia (CAP)-related sepsis and found two different subtypes of the characteristic sepsis response^6^. Abundant evidence suggests that N6-methyladenosine (m6A) is involved in a variety of biological processes, especially in sepsis and immune diseases; however, its role in the classification of sepsis remains unclear^7,8^. m6A is the most common epigenetic modification in higher eukaryotes. Through the abnormal expression of "writer"-, "eraser"- and "reader"-related factors, m6A can dynamically and reversibly regulate many complex cellular processes, such as RNA processing, transportation, localization, translation and degradation^9,10^.

In sepsis, overactivation of the immune response leads to overproduction of various proinflammatory cytokines and induces cell damage. In recent years, m6A modification has not only been shown to be involved in tumor growth and the immune response to viral infection but has also been indicated to be related to several inflammatory diseases^11–13^. For instance, the writer-related protein METTL3 mediates increased expression of inflammatory cytokines such as IL-6 and IL-8 and activation of NF-κB signaling^14^. Moreover, the eraser-related protein ALKBH5 upregulates MALAT1 expression through demethylation and participates in the production of inflammatory cytokines in LPS-stimulated HK-2 cells^15^.

Given the available research results, m6A regulators have a major function in the development of sepsis^16^. However, the roles of m6A regulators in the diagnosis and classification of sepsis are rarely studied. Therefore, we assessed the expression level of 26 m6A regulators and the immune cell content in sepsis patients via the Gene Expression Omnibus (GEO) database. Moreover, random forest (RF) and clustering analyses were used to predict the clinical condition, immune characteristics and inflammation level of sepsis patients.

## Materials and methods

### Data collection and processing

Gene expression data for sepsis patients were obtained from the GSE65682 dataset in the Gene Expression Omnibus (GEO) database. This dataset contains data for 760 patients with sepsis and 42 healthy controls. Twenty-six m6A regulators, namely, 9 writers, 15 readers and 2 erasers, were identified by previous studies, and these genes are shown in Table S1.

### Differentially expressed genes (DEGs) between sepsis patients and healthy controls

The "limma" software package in R software was used to analyze the differentially expressed genes (DEGs) between sepsis patients and healthy controls with thresholds of |log2FC|≥ 1 and FDR ≤ 0.05^17^. Pearson correlation analysis was conducted to reveal the relationships among m6A-related genes^18^.

### PPI network construction

A PPI network was constructed with the STRING database to reveal the molecular mechanism involved in sepsis. The above DEGs were imported into the STRING database; the study species was defined as “*Homo sapiens*”, and the rest of the parameters were set to the default values^19^.

### Evaluation of immune cells

CIBERSORT is a typical deconvolution tool for analyzing an immune cell expression matrix based on linear support vector regression, which quantifies the proportions of infiltrating immune cells by determining the expression of marker genes. In our study, we combined the expression of 22 immune cell marker genes with transcriptome data from all sepsis patients to obtain immune cell scores via CIBERSORT^20^.

### Random forest (RF) and support vector machine (SVM) model construction

The “Randomforest” package was used to build the RF model. A large number of classification trees were randomly generated, and the m6A regulator classification results for each tree were iteratively scored to obtain the classification outcome^21^. Finally, the classification results of all single trees were comprehensively assessed, and the “Caret” package was used to rank the importance of m6A regulators in the RF model^22^. The R software e1071 package was used to construct the SVM model. The hub gene of the selected module was taken as the independent variable, and the corresponding support vector was first found. Then, an optimal classification hyperplane that not only met the classification requirements but also ensured the classification accuracy and maximized the blank area on both sides of the hyperplane, was found^23^. ROC analysis was performed to determine the predictive accuracy of the two models by using the pROC package, and the area under the curve (AUC) was calculated^24^.

### Construction and validation of the Predictive Nomogram

The nomogram was generated by R software with the “rms” package based on the expression level of the top 10 important m6A regulators, and decision curve analysis (DCA) was conducted to assess the predictive value of the nomogram^25^.

### Unsupervised clustering analysis

Based on the expression profiles of the m6A regulators, we performed unsupervised clustering analysis of the sepsis patient samples using the "ConsensusClusterPlus" software package and divided the samples into two m6A clusters^26^. The following parameters were set: max cluster number (maxK) = 9, proportion of items to sample (pItem) = 0.8, proportion of features to sample (pFeature) = 1, cluster algorithm (clusterAlg) = hc/hierarchical, and distance = spearman. The above process was repeated 1,000 times to ensure the consistency of the classification. Principal component analysis (PCA) was used for dimensionality reduction^27^. Pearson correlation analysis was used to reveal the correlations of m6A regulator expression and immune cells.

### DEGs between the two m6A clusters and functional analysis

The "limma" software package was used to analyze the differentially expressed genes (DEGs) between the two m6A clusters with thresholds of |log2FC|≥ 1 and FDR ≤ 0.05. We used "clusterProfiler" to perform GO (Gene Ontology) and KEGG (Kyoto Encyclopedia of Genes and Genomes) functional enrichment analyses to explore the potential biological functions of these DEGs^28^.

### Construction of the m6A gene signature

The PCA algorithm was used to calculate the m6A score of each sample to quantify m6A patterns. The m6A score was calculated as Σ(Expi * PCi), where PCi and Expi represent the principal component and expression level of each gene, respectively^29^.

### Assessment of immune infiltration

The single-sample gene set enrichment analysis (ssGSEA) algorithm was used to estimate immune infiltration in each sepsis sample, and the enrichment score was used to reveal the enrichment degree of each immune cell^30^.

### Statistical analysis

All analyses were performed in R software (version 4.1.0)^31^. Spearman and distance correlation analyses were performed to estimate correlation coefficients between the expression of m6A regulators and infiltrating immune cells. The Wilcoxon test was performed to analyze variations between two groups. A ROC curve was generated to verify the validity of the model. P < 0.05 was considered statistically significant.

## Results

### Transcriptional alterations in m6A regulators in sepsis

**Figure 1:**
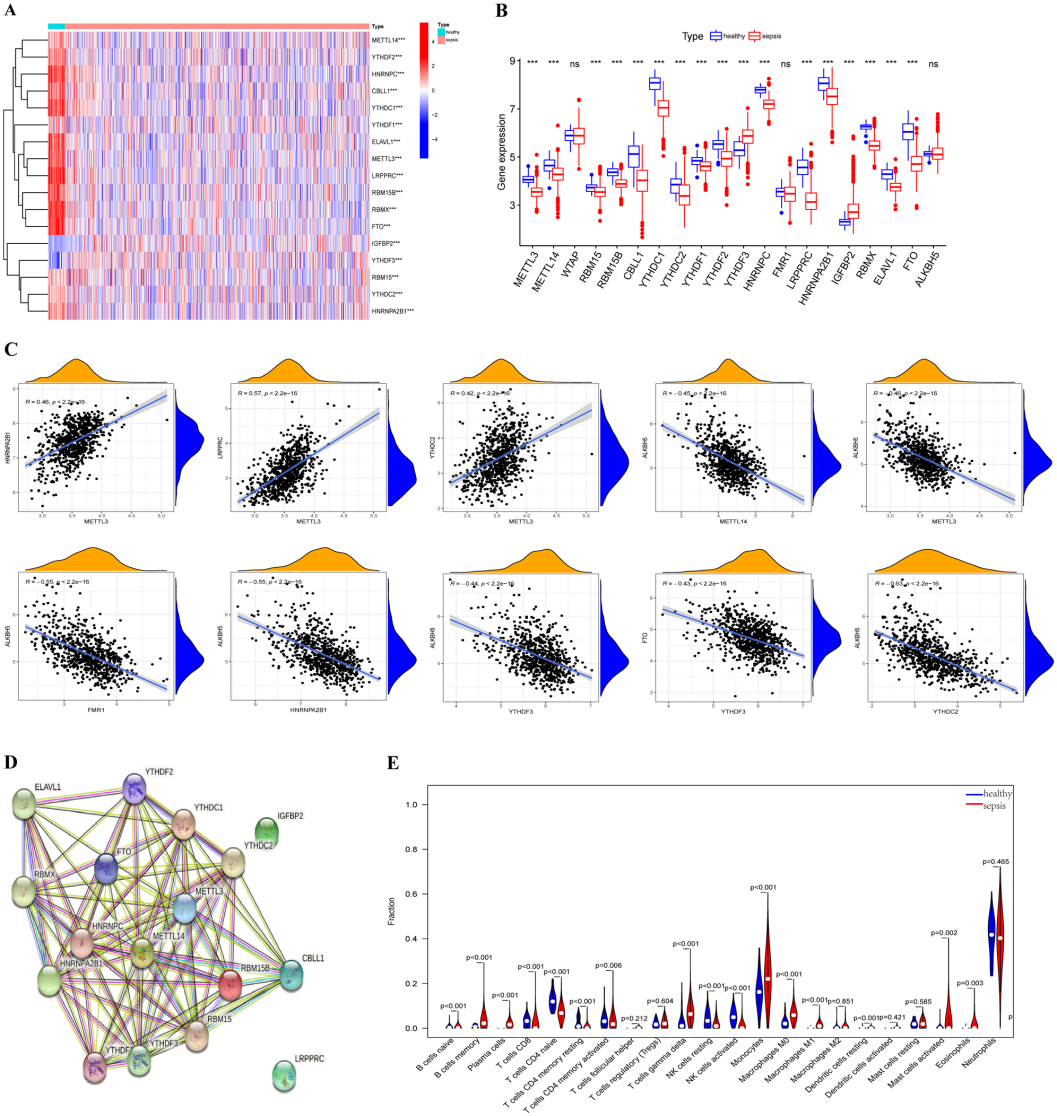
Landscape of and correlations between 26 m6A regulators in sepsis. Heatmap (**A**) and box plot (**B**) of 26 m6A regulators in sepsis patients and healthy controls. (**C**) The correlations between the 26 m6A regulators. (**D**) PPI network of the 17 differentially expressed m6A regulators. (**E**) The differences in 22 types of infiltrating immune cells between patients with sepsis and healthy controls. *ns* not significant; ***P < 0.001.

To identify the differentially expressed genes encoding m6A regulators in sepsis patients, differential expression analysis was performed with the “limma” package to analyze 802 blood samples from the GSE65682 dataset, namely, 760 from patients with sepsis and 42 from healthy controls. The heatmap revealed that the expression of 17 m6A regulators was significantly different between the patients with sepsis and the healthy controls (Fig. 1A). We found that the expression of METTL3, METTL14, RBM15, RBM15B, CBLL1, YTHCD1, YTHDC2, YTHDF1, YTHDF2, YTHDF3, HNRNPC, LRPPRC, HNRNPA2B1, RBMX, ELAVL1 and FTO was significantly lower in the patients with sepsis, while IGFBP2 expression was significantly higher in the patients with sepsis. However, we observed no significant differences in WTAP, FMR1 and ALKBH5 expression (Fig. 1B). Next, we explored the relationships among the m6A regulators, and correlation analysis was performed (Fig. 1C). The results showed that METTL3 expression was significantly positively correlated with the expression of HNRNPA2B1, LRPPRC and YTHDC2. In addition, ALKBH5 expression was significantly negatively related to the expression of METTL3, METTL14, FMR1, HNRNPA2B1, YTHDF3, and YTHDC2, and FTO expression was significantly negatively associated with the expression of FTO. The STRING database was used to construct a PPI network containing these 17 m6A regulators (Fig. 1D). Moreover, we analyzed the differences in 22 infiltrating immune cells between patients with sepsis and healthy controls. The results demonstrated that the proportions of naïve B cells, memory B cells, plasma cells, gamma delta T cells, monocytes, M0 macrophages, M1 macrophages, resting dendritic cells, activated mast cells and eosinophils were higher in sepsis patients. In contrast, the proportions of CD8 + T cells, naïve CD4 + T cells, resting memory CD4 + T cells, acting memory CD4 + T cells, resting NK cells and activated NK cells in sepsis patients were lower than those in healthy controls (Fig. 1E). The m6A regulator expression levels were significantly different between healthy controls and sepsis patients, and there were different correlations between m6A regulators in sepsis patients.

### Construction of the random forest (RF) model and support vector machine (SVM) model

**Figure 2:**
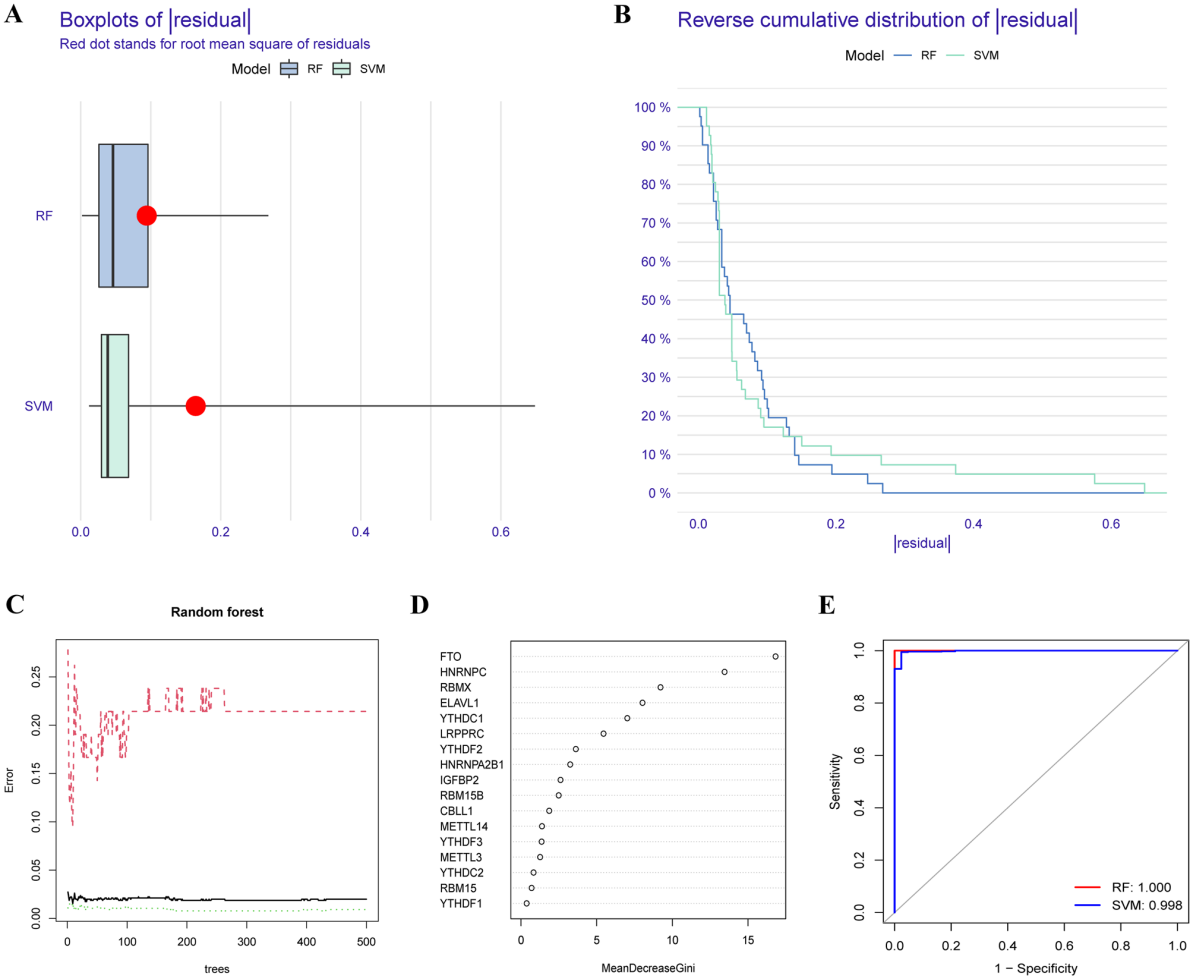
Construction of the random forest (RF) model and support vector machine (SVM) model. The residual (**A**) and reverse cumulative distribution (**B**) for the RF model and SVM model. (**C**) The effect of the number of decision trees on the error rate. (**D**) The importance of the 17 differentially expressed m6A regulators in the RF model. (**E**) ROC analysis of the predictive accuracy of the two models.

To establish a diagnostic m6A-related gene signature, RF and SVM models were constructed separately. The boxplots showing the |residual| and reverse cumulative distribution of |residual| values revealed that the residual distribution for the RF model was lower than that for the SVM model (Fig. 2A,B). Based on the above results, the RF model was considered a more appropriate model to predict the occurrence of sepsis. Then, according to the overview of the relationship between the model error and the number of decision trees, 500 trees were selected as the variables in this model, which presented a stable error probability (Fig. 2C). Next, we ranked the m6A-related genes by assessing their importance, and the results indicated that FTO, HNRNPC, and RBMX were the 3 most important genes (Fig. 2D). Furthermore, ROC analysis was used to compare the accuracy of the two models, and the AUC value for the RF model was higher than that for the SVM model, indicating that the RF model had higher accuracy (Fig. 2E). The RF model had the smallest residual distribution and the highest AUC value, indicating that it was the most appropriate training model; thus, we choose the RF model for further analysis.

### Construction of a predictive nomogram

**Figure 3:**
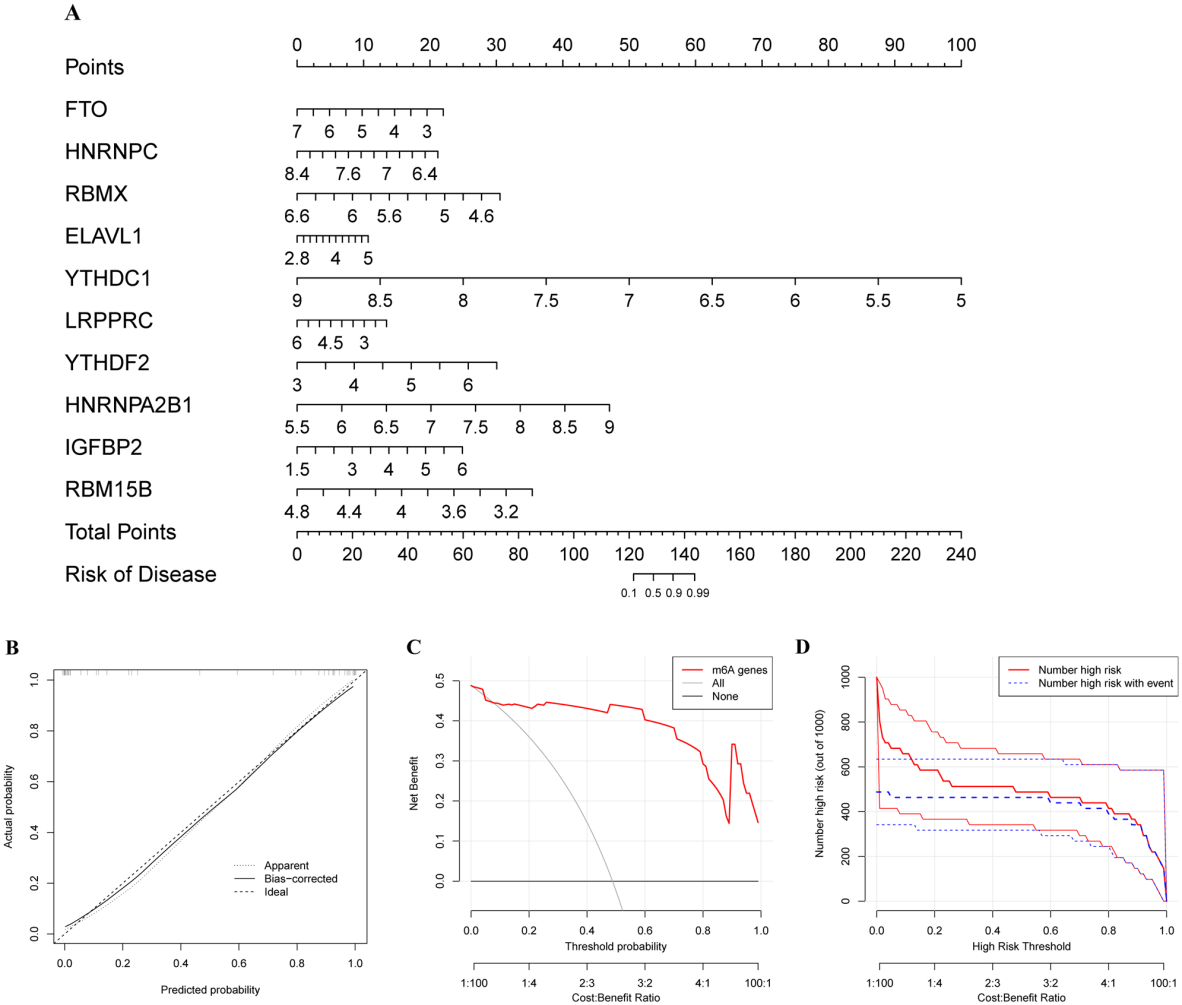
Construction and validation of the predictive nomogram. (**A**) Establishment of the predictive nomogram consisting of 10 m6A regulators. (**B**) The calibration curve was used to validate the predictive ability of the nomogram. (**C**) Decision based on the nomogram may benefit patients with sepsis. (**D**) The clinical impact curve was constructed based on the clinical impact of the nomogram.

To better evaluate the risk of sepsis, a nomogram was constructed to combine the 10 recommended m6A regulators (Fig. 3A). In this predictive nomogram, the expression levels of FTO, HNRNPC, RBMX, YTHDC1, LRPPRC, and RBM15B were negatively associated with the risk score of patients with sepsis and were regarded as protective factors in sepsis. A calibration curve was utilized to determine the predictive accuracy of the nomogram (Fig. 3B). DCA indicated that patients with sepsis could benefit from predictive decisions based on this nomogram, as the decision curve constructed with the m6A regulator-related model indicated the greatest benefit (Fig. 3C). Moreover, the clinical impact curve confirmed that this nomogram was reliable and robust (Fig. 3D).

### Subtype analysis of m6A regulators

**Figure 4:**
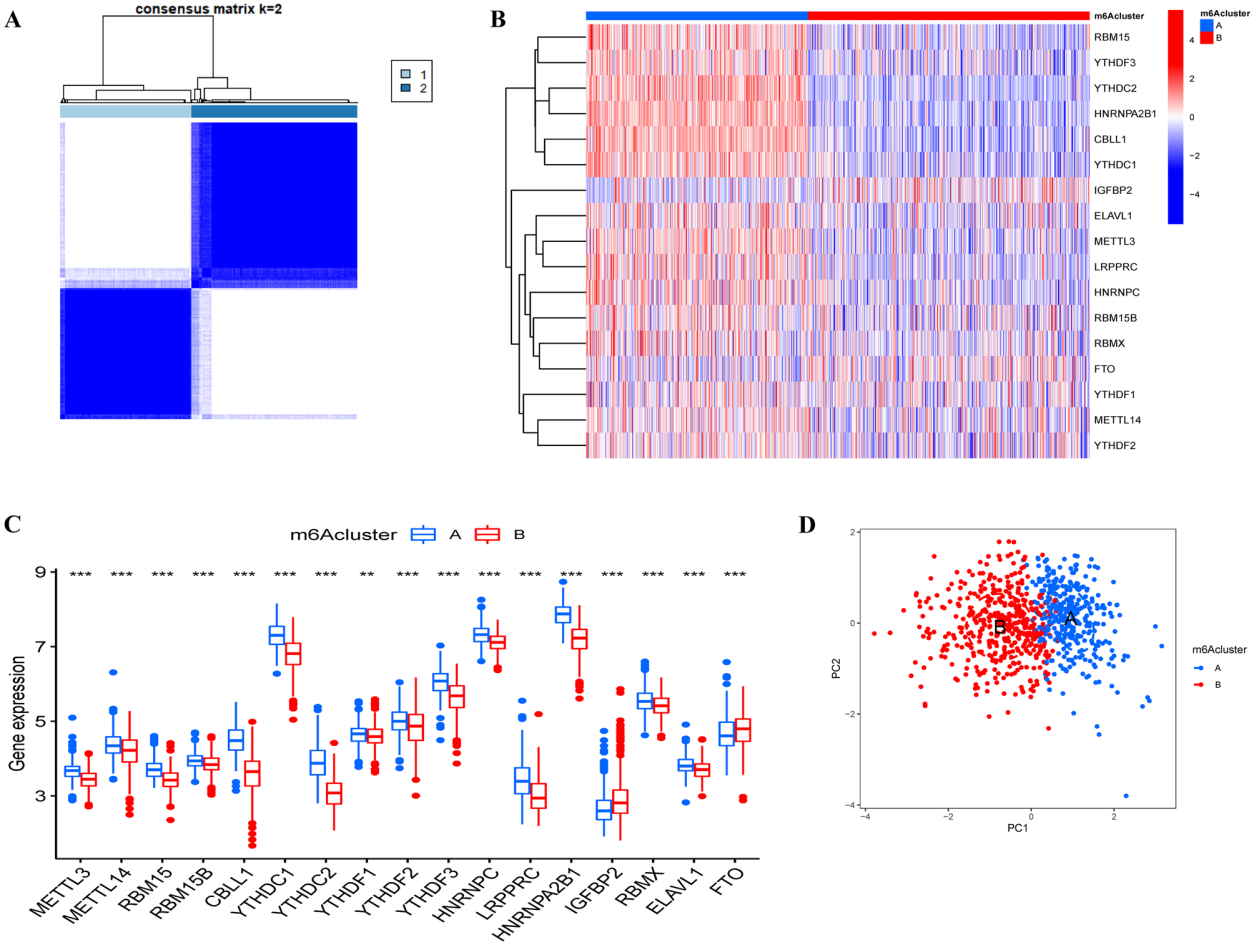
Consensus clustering analysis of the 17 differentially expressed m6A regulators in sepsis. (**A**) When *k* = 2, the consensus clustering analysis was the most reliable. The heatmap (**B**) and box plot (**C**) revealed the differential expression of the 17 m6A regulators between cluster A and cluster B. (**D**) Principal component analysis (PCA) revealed a significant difference between cluster A and cluster B. **P < 0.01; ***P < 0.001.

**Figure 5:**
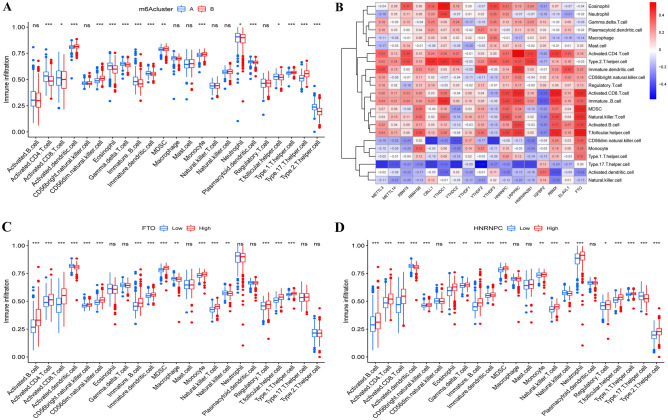
Single-sample gene set enrichment analysis (ssGSEA) between cluster A and cluster B. (**A**) Different abundances of infiltrating immune cells between cluster A and cluster B. (**B**) The associations of the 17 differentially expressed m6A regulators and infiltrating immune cells. (**C**,**D**) Differential immune cell infiltration in samples with different expression levels of FTO and HNRNPC. *ns* not significant; *P < 0.05; **P < 0.01; ***P < 0.001.

To further determine the relationships between the expression patterns of m6A regulators and sepsis subtypes, a consensus clustering method was used to reveal the role of the m6A-related genes, and when *k* = 2, the sepsis patients were divided into two subgroups (m6A clusters A and B), and the correlation between the two groups was higher than that for other values of *k* (Fig. 4A). The heatmap shows the differences in m6A-related gene expression between the two groups (Fig. 4B). As shown in Fig. 4C, METTL3, METTL14, RBM15, RBM15B, CBLL1, YTHDC1, YTHDC2, YTHDF1, YTHDF2, YTHDF3, HNRNPC, LRPPRC, HNRNPA2B1, RBMX and ELAVL1 were upregulated in m6A cluster A, whereas IGFBP2 and FTO were upregulated in m6A cluster B. The PCA results showed that the two m6A clusters could be significantly distinguished (Fig. 4D). Furthermore, single-sample gene set enrichment analysis (ssGSEA) was utilized to quantify the differences in infiltrating immune cells between the two m6A clusters, and the correlations between the expression of important m6A-related genes and immune cells were revealed. There was a significant difference in immune activity between the two m6A clusters, and s higher abundance of immune cells was observed in m6A cluster A (Fig. 5A). Accordingly, we found that the expression of HNRNPC, LRPPRC, RBMX, and FTO was positively correlated with the presence of multiple types of immune cells, while YTHDF3 and IGFBP2 expression was negatively associated with numerous types of immune cells (Fig. 5B). In addition, the most significant negative correlation was found between T helper 17 cells and m6A-related genes, as shown in Fig. 5B. Next, we investigated differences in immune cells in patients with high expression or low expression of FTO and HNRNPC (Fig. 5C,D). We found greater enrichment of immune cells in sepsis patients with higher FTO and HNRNPC expression, which indicated that sepsis patients with higher FTO and HNRNPC expression had a more positive immune response to sepsis.

### Construction of the m6A gene signature

**Figure 6:**
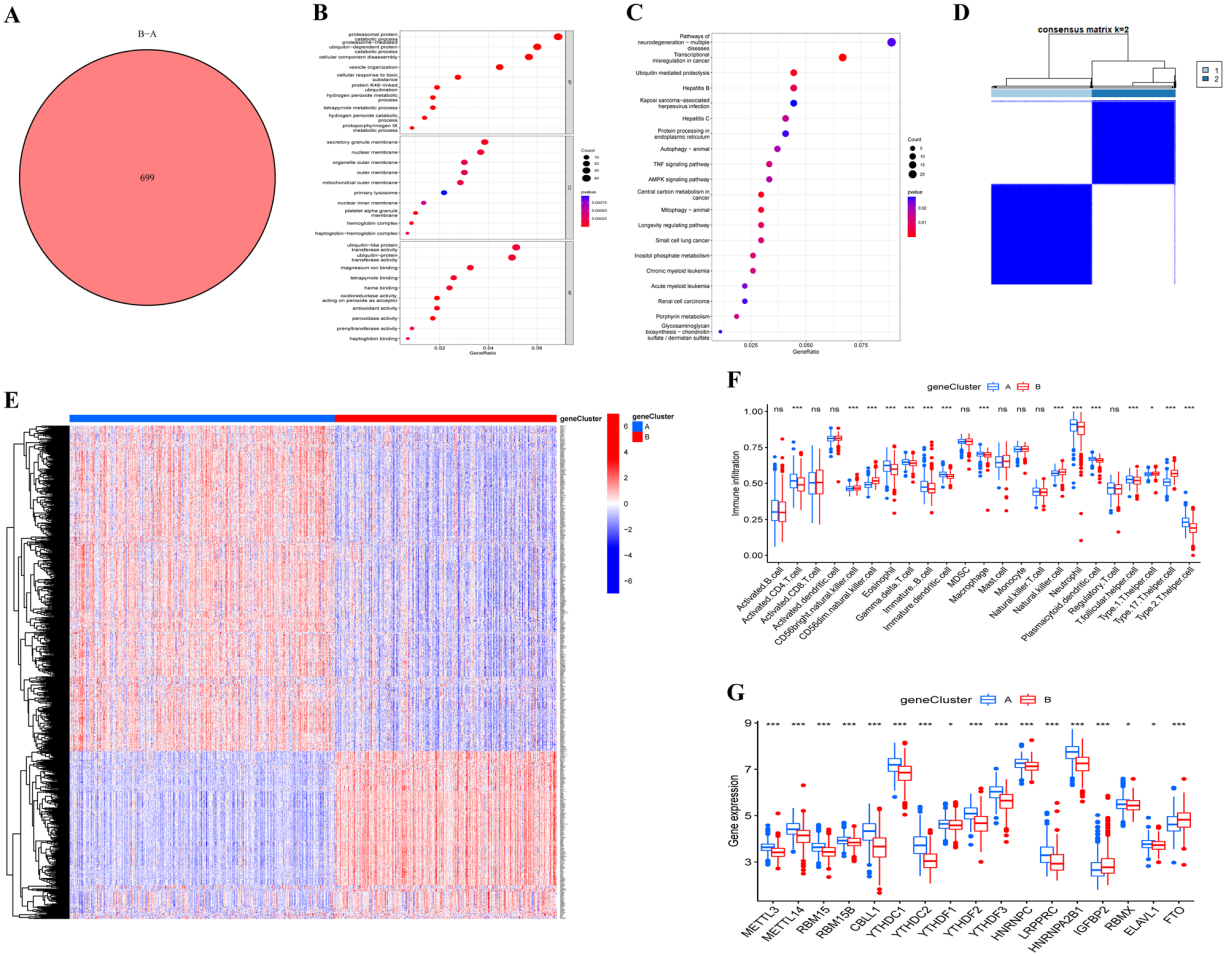
Consensus clustering analysis of the differentially expressed genes (DEGs) between cluster A and cluster B. (**A**) A total of 699 DEGs were identified between cluster A and cluster B. Functional annotation of the 699 DEGs, including Gene Ontology analysis (**B**) and Kyoto Encyclopedia of Genes and Genomes pathway analysis (**C**). (**D**) Gene consensus clustering analysis of 699 DEGs for *k* = 2, with identification of two gene clusters. (**E**) The distribution of the 699 DEGs between gene cluster A and gene cluster B. (**F**) The difference in immune cell infiltration between gene cluster A and gene cluster B. (**G**) The differential expression of the 17 m6A regulators between gene cluster A and gene cluster B. *ns* not significant; *P < 0.05; ***P < 0.001.

To investigate the underlying biological activity of the m6A-related cluster, we identified 699 m6A-related cluster-associated DEGs between m6A cluster A and m6A cluster B (Fig. 6A). Next, functional analysis was conducted with the “limma” package to reveal the roles of the DEGs. In the biological process (BP) category, the DEGs were mainly involved in proteasomal protein catabolic process, proteasome-mediated ubiquitin-dependent protein catabolic process and cellular component disassembly. DEG enrichment in the cellular component (CC) category was mainly concentrated in the terms secretory granule membrane, nuclear membrane and organelle outer membrane. The molecular functions (MF) of the DEGs were mainly related to ubiquitin-like protein transferase activity, ubiquitin-protein transferase activity and magnesium ion binding (Fig. 6B). In addition, KEGG analysis demonstrated enrichment of the DEGs in pathways of neurodegeneration-multiple diseases, transcriptional misregulation in cancer and ubiquitin-mediated proteolysis (Fig. 6C). To study the specific regulatory mechanism, the patients were divided into gene clusters (gene cluster A and B) according to DEGs by the consensus clustering method (Fig. 6D). The heatmap shows the differences in the expression of 699 m6A-related cluster-associated DEGs between gene cluster A and B, with 237 upregulated and 462 downregulated genes in gene cluster A (Fig. 6E). Additionally, differences in immune cells between the two gene clusters were identified by ssGSEA (Fig. 6F). The m6A-related gene clusters demonstrated substantial differences in m6A-related gene expression, as expected from the m6A subgroups (Fig. 6G).

### Correlations between m6A subgroups and cytokines

**Figure 7:**
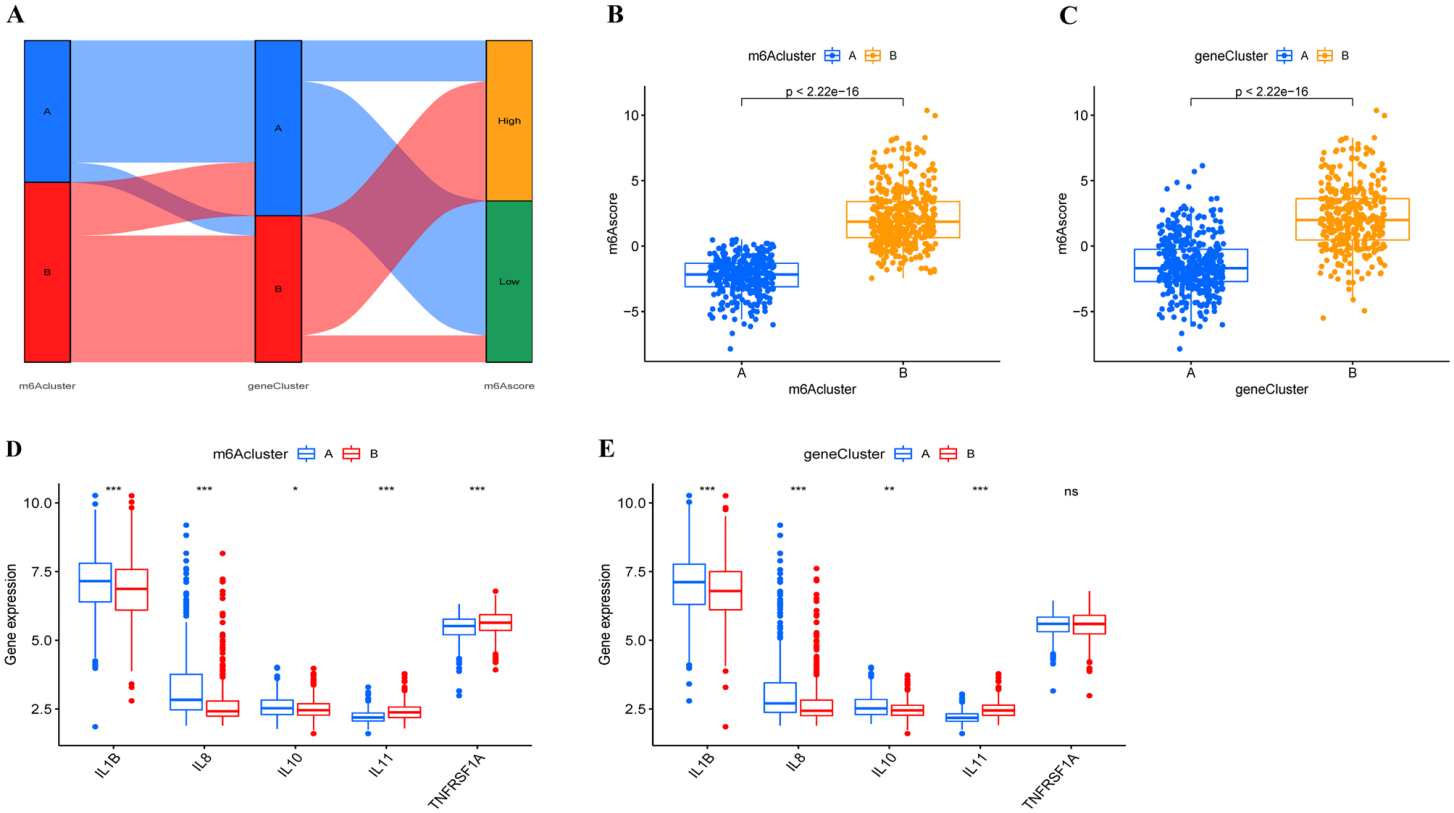
The role of the m6A subtype in distinguishing sepsis patients. (**A**) Sankey diagram displaying the distribution of sepsis patients in the two m6A clusters, two gene clusters and two m6A score groups. The difference in m6A scores in the two m6A subtypes, including m6A clusters (**B**) and gene clusters (**C**). The expression levels of inflammatory factors (IL-1β, IL-8, IL-10, IL-11 and TNFRSF1A) in the two m6A subtypes, including m6A clusters (**D**) and gene clusters (**E**). *ns* not significant; *P < 0.05; **P < 0.01; ***P < 0.001.

According to the expression of m6A-related genes, principal component analysis (PCA) was used to determine a score for each sepsis patient, which was defined as the m6A score. The Sankey diagram shows the distribution of the two m6A clusters, two gene clusters and two m6A scores (Fig. 7A). In addition, we found substantial differences in the m6A score between the m6A clusters and gene clusters (Fig. 7B,C), with a higher m6A score in m6A cluster B and gene cluster B, revealing the correlation of the m6A score and immune activation. Throughout the course of sepsis, an imbalance in inflammatory factors is the key to sepsis pathogenesis^32^. Further research was conducted to reveal the associations between m6A patterns and inflammatory factors, including interleukin (IL)-1β, IL-8, IL-10, IL-11 and TNF receptor superfamily member 1A (TNFRSF1A), in sepsis. The results showed that the expression levels of IL-1β, IL-8, and IL-10 were significantly higher in m6A cluster A and gene cluster A, while IL-11 expression was significantly upregulated in m6A cluster B (Fig. 7D, E). The above results indicated that two m6A-related subgroups (m6A cluster and m6A gene cluster) can help us to predict the levels of inflammatory factors and risk score of disease in sepsis patients.

## Discussion

Sepsis is life-threatening organ failure caused by an uncontrolled host response to infection^2^. The comprehensive treatment of sepsis has been further developed, and the mortality of sepsis has been declining, but problems such as infection with multidrug-resistant bacteria, coagulation dysfunction, and gastrointestinal dysfunction have increased the difficulty of treatment^33,34^. Emerging studies have demonstrated that m6A-related genes are involved in multiple biological processes in sepsis^35,36^. However, the potential role of m6A regulators in sepsis has rarely been studied.

In this study, we revealed the alterations in and interactions of m6A regulators at the transcriptional level in sepsis. In addition, we found significant differences in the proportions of immune cells between patients with and without sepsis. These results showed that m6A regulators and immune cells may have important links with the occurrence and development of sepsis. Furthermore, we compared two predictive models based on the expression levels of m6A regulators, and significant advantages of the RF model were observed. Therefore, a predictive nomogram was constructed with the 10 most important m6A regulators identified by the RF model, which provides a new research direction for further analysis of patients with sepsis. The nomogram provides us with an intuitive and user-friendly interface to predict the risk of sepsis. We can collect blood samples from patients and test the expression of the 10 genes based on the 10 identified biomarkers. Each gene has a score based on its expression level, and the sum of all the scores provides a total score that can be used to predict the incidence of sepsis.

To build an m6A regulator-related signature, random forest (RF) and support vector machine (SVM) models were developed. The RF is a learning algorithm that combines different decision trees. The RF model consists of independent decision trees. Each tree is generated based on random samples. Each decision tree learns and predicts independently, and the final result is based on the average value of all trees^1^. SVM is a discriminant classifier that uses a classification hyperplane to define classifications. Labeled training samples are used to train the model, and then the optimal hyperplane output is used to classify the test samples^2^. In the comparison between the RF model and the SVM model, the established RF model had the smallest residual distribution and the highest AUC value and was thus the most appropriate training model.

According to the above research results, FTO, HNRNPC, RBMX, ELAVL1, YTHDC1, LRPPRC, YTHDF2, HNRNPA2B1, IGFBP2 and RBM15B were the 10 most important genes and were included in the construction of the sepsis nomogram. Multiple omics studies have shown that the FTO level is significantly reduced in the serum of sepsis-associated encephalopathy (SAE) patients with higher levels of inflammation and reduced diversity of the gut microbiome^37^. Similarly, the expression of FTO is decreased in the myocardium of mice with lipopolysaccharide (LPS)-induced endotoxemia, and FTO knockdown in cardiomyocytes mimics the LPS-induced effect^38^. The urokinase-type plasminogen activator (uPA) receptor (uPAR) is upregulated during acute lung injury caused by sepsis and promotes the development of pulmonary edema and the expression of proinflammatory cytokines^39^. The interaction of HNRNPC with the uPAR mRNA coding region and 3′ untranslated region determines the posttranscriptional stability of uPAR mRNA^40^. In addition, IGFBP2 has been proven to be upregulated in patients with sepsis and mice with high-dose nandrolone-induced septic shock^41,42^, consistent with our study. However, relationships between other candidate m6A regulators and sepsis have not been reported, and further studies are needed to show the functions of these m6A regulators. To evaluate the characteristics of m6A regulator patterns in classifying immune features, all sepsis patients were divided into two subgroups by consensus clustering analysis. Based on the similarity of m6A regulator expression levels and the fuzzy clustering proportional membership, it was found that when k = 2, the cluster had the best stability. Therefore, patients with sepsis were assigned to two clusters: cluster 1 and cluster 2. Then, the differences in m6A regulator expression and immunity between the two clusters were compared.

Previous studies have suggested that the initial stage of sepsis is a period of excessive inflammatory response, which can be followed by an immunosuppressive period as the disease develops^43^. The excessive activation of innate immunity leads to the persistence of an excessive inflammatory response, which leads to dysfunction of the corresponding organs^44^. Therefore, systemic analysis of host immunity in sepsis can provide a theoretical basis for better diagnosis and treatment of sepsis. We identified 17 DEGs between patients with and without sepsis and then performed unsupervised clustering analysis to divide the patients into two m6A subtypes and two gene clusters. Furthermore, we found similar CD4 + T-cell infiltration level trends in both m6A cluster A and gene cluster A, which helped to predict the clinical features of sepsis. In addition, we quantified the m6A subtypes by calculating m6A scores, and both m6A cluster A and gene cluster A had lower m6A scores. Throughout the course of sepsis, an imbalance in the inflammatory response is the key to sepsis pathogenesis. The host’s acute response to an invading pathogen usually results in macrophages engulfing the pathogen and producing diverse proinflammatory mediators that trigger a cytokine storm and activate the innate immune system^45^. Both the m6A clusters and gene clusters showed similar trends in inflammatory factor levels.

However, this study still has some limitations. First, the conclusions were drawn from analysis of a single dataset in a public database, and there is a lack of multiple clinical data sources and experimental studies to verify the relationship between m6A regulators and sepsis. Because it may be more difficult to collect serum samples from healthy patients in the clinic, the number of healthy samples in this dataset cannot match the number of samples from sepsis patients, which may also lead to meaningless or false-positive results in the differential expression analysis between the two groups. To clarify sepsis more clearly, we can add more clinical details to the nomogram. It is difficult to promote the clinical application of nomograms, but with the development of next-generation sequencing technology and more convenient and rapid sequencing chips, this problem may be solved in the future. In addition, due to data limitations, our analysis lacked a large clinical cohort for validation.

## Conclusion

Our comprehensive analysis of m6A regulators in patients with sepsis revealed the regulatory mechanisms that affect the characteristics of immune cell infiltration and the levels of inflammatory factors, and we constructed a nomogram to determine their value in predicting the risk of sepsis. Based on the expression of 17 significant m6A regulators, two m6A subtypes were identified, and cluster B may be obviously correlated with sepsis.

## Supplementary Information

Supplementary Table S1.

## Data Availability

The dataset generated and analyzed during the current study is available in the Gene Expression Omnibus (GEO) under accession number GSE65682 (https://www.ncbi.nlm.nih.gov/geo/query/acc.cgi?acc=GSE65682).
