# Supplementary material for: Diagnostic, clustering, and immune cell infiltration analysis of m6A regulators in patients with sepsis
Source: Sci Rep. 2023 Feb 13;13:2532. doi: 10.1038/s41598-022-27039-4 (PMC9925440; doi:10.1038/s41598-022-27039-4)
Supplement: Supplementary file 1 — Supplementary Table S1. [file 41598_2022_27039_MOESM1_ESM.docx]

**Table S1 Summary of 26** RNA N6-methladenosine (m6A) regulators

| Gene | Type |
| --- | --- |
| METTL3 | writers |
| METTL14 | writers |
| METTL16 | writers |
| WTAP | writers |
| VIRMA | writers |
| ZC3H13 | writers |
| RBM15 | writers |
| RBM15B | writers |
| CBLL1 | writers |
| YTHDC1 | readers |
| YTHDC2 | readers |
| YTHDF1 | readers |
| YTHDF2 | readers |
| YTHDF3 | readers |
| HNRNPC | readers |
| FMR1 | readers |
| LRPPRC | readers |
| HNRNPA2B1 | readers |
| IGFBP1 | readers |
| IGFBP2 | readers |
| IGFBP3 | readers |
| RBMX | readers |
| ELAVL1 | readers |
| IGF2BP1 | readers |
| FTO | erasers |
| ALKBH5 | erasers |
